# Current News

**Published:** 1902-09

**Authors:** 


### Current News.

### NATIONAL ASSOCIATION OF DENTAL EXAMINERS.

At the meeting of the National Association of Dental Examiners, held at Niagara Falls, July 28 to 31, 1902, the following officers were elected:

President, Charles A. Meeker, Newark, N. J.; Vice-President from the West, Burton Lee Thorpe, St. Louis, Mo.; Vice-President from the East, J. A. Libbey, Pittsburg, Pa.; Vice-President from the South, J. A. Hall, Collinsville, Ala.; Secretary, Joseph P. Root, Kansas City, Kan.

*Committee on Colleges.*—C. C. Chittenden, Madison, Wis.; J. A. Hall, Collinsville, Ala.; H. J. Burkhart, Batavia, N. Y.

*Committee on Conference.*—G. E. Mitchell, Haverill, Mass.; J. G. Reid, Chicago, Ill.; J. A. Libbey, Pittsburg, Pa.

*Membership Committee.*—W. M. Darwood, Omaha, Neb.; P. J. Heffern, Pawtucket, R. I.; J. E. Weirrick, St. Paul, Minn.

*State's Advisory Committee.*—William Jarvie, Brooklyn, N. Y.; F. A. Shotwell, Rogersville, Tenn.; H. J. Allen, Washington, D. C.

*Committee for promoting Relations with Foreign Countries.*—William Carr, New York City; G. W. Pelzer, Great Falls, Mont.; H. W. Campbell, Suffolk, Va.; R. H. Jones, Winston, N. C.

*Committee on Contracts and Accommodations.*—J. Allen Osmun, Newark, N. J.

